# Selecting graduates for the interns’ award by using multisource feedback process: does it work?

**DOI:** 10.1186/s13104-017-2848-6

**Published:** 2017-10-30

**Authors:** Kathryn Strachan, Sameer Otoom, Amal AL-Gallaf, Ahmed Al Ansari

**Affiliations:** 1 RCSI Bahrain, P.O. Box 15503, Adliya, Bahrain; 2 Training and Education Department, Bahrain Defense Force Hospital, Off Waly Alahed Avenue, West Riffa, P. O. Box 28743, Riffa, Bahrain; 3 Arabian Gulf University, Manama, Bahrain

**Keywords:** MSF, Interns, Professionalism, Communication, Collaboration

## Abstract

**Background:**

The purpose of this study is to find a reliable method for choosing graduates for a higher-education award. One such method that has achieved notable popularity is known as multisource feedback. Multisource feedback is assessment tool that uses evaluations of different groups and includes both physicians and non-physicians. It is useful for assessing several domains, including professionalism, communication and collaboration, and therefore is a valuable tool for providing a well-rounded selection of the top interns for postsecondary awards. 16 graduates in Royal College of Surgeons in Ireland-Medical University of Bahrain (RCSI Bahrain) responded to an invitation to participate in the student award, which was conducted by the using the multisource feedback process. 5 individuals from different categories (physicians, nurses, and fellow students), rated each participant in this study. A total of 15 individuals were the proposed number for rating. The ratings were calculated using mean and standard deviation, and the award went to the one of the top score out of the 16 participants. Reliability and internal consistency was calculated using Cronbach’s coefficient, and construct validity was evaluated using factor analysis.

**Results:**

16 graduates participated in the Royal College of Surgeons in Ireland-Bahrain interns’ award based on the multisource feedback process, giving us a 16.5% response rate. The instrument was found to be suitable for factor analysis and showed 3 factor solutions representing 79.3% of the total variance. Reliability analysis using Cronbach’s α reliability of internal consistency indicated that the full scale of the instrument had high internal consistency (Cronbach’s α 0.98).

**Conclusion:**

This study confirmed our hypothesis, finding multisource feedback to be a process for choosing the most suitable graduates for interns’ awards that is both reliable and valid. Unfortunately, there were low response rate, which could mean that multisource feedback is not a realistic way to bring most students into the process.

## Background

Although it is a difficult task, finding a reliable method for choosing graduates for a higher-education award is far from impossible. The reason for this challenge is that the selection method should be realistic, acceptable, valid, reliable, and makes a positive difference for educational outcomes [1]. Such a reliable evaluation method will help to select the best candidates and will show the strengths and weaknesses of the graduates [2]. It is extremely important to prove an in-depth evaluation of medical graduates, since they are just beginning their careers in the profession. In addition to selecting the best candidates for the university award, the feedback received from these evaluations will improve educational outcomes by showing areas for individuals to focus on to strengthen their future performance [3].

Multisource feedback (MSF), which is a very popular process also known by some researchers as the 360° evaluation, is an evaluation process in which various raters fill out surveys to evaluate their medical peers and colleagues. This evaluation process provides feedback from individuals who are not the attending and/or supervising physicians [4]. This type of assessment uses raters from a variety of groups who interact with trainees [5, 6].

The multisource feedback (MSF) process is seen as a particularly effective framework for evaluating physicians regarding their interactions and relationships [7]. MSF focusses on assessing different domains such as clinical skills, communication, professionalism, collaboration, and patient management [8, 9].

Since the MSF process is an effective, reliable, valid, and streamlined method for evaluation [10–12], we decided to use it in this study as the primary selection criteria for potential candidates to be awarded and labeled as the top university graduates. To our knowledge, this method has not previously been used to select candidates for students’ awards in higher education. The aims of this study therefore were: (1) to select the best candidates among the graduates based on the MSF evaluation, and (2) to analyze the effectiveness, validity, and reliability of MSF as a process for selecting graduates who are the best fit for the university award.

## Methods

We invited all of our graduates for this year—97 students—to participate in the interns’ award. These potential participants were students who had completed medical school and began year-long internship rotation at different hospitals. All the interns who had finished their medical programme at RCSI Bahrain and started their first-year internship rotation were contacted by email. They were informed of the award competition and its purpose, requirements, selection criteria, and a guide for implementing the MSF process. Interns were sent an email with a form that consisted of three tables to be completed by eight nominated colleagues from each of the three different categories: interns, chief resident/consultant, and co-workers/nurses. In addition to these three categories, evaluation forms and a self-evaluation form were expected to be completed.

The nomination form entailed some details about the raters, including: position, job title, department, and email address. Only sixteen interns were interested in applying for the award and each submitted their nominees’ list to an independent administrative team at RCSI Bahrain. The independent administrative team sent the evaluation forms to the raters and requested them to complete the forms and send them back. Each evaluator was given a month to complete and send the forms by e-mail or in person to the administrative team at RCSI-Bahrain. Given a sufficient period of time for completing the evaluation forms, raters who did not submit their forms were contacted, as a reminder, by means of a second email through the administrative team. The independent administrative team was responsible for distributing the instruments electronically, collecting them, anonymizing the forms using a number code for each intern, and inputting all data into Microsoft Excel Worksheet.

This study had three groups of people who rated the candidates: nurses, physicians, and student colleagues. The candidates selected eight individuals from each of these three categories, and the researchers randomly selected five out of these eight. Five members of each of these three groups were therefore responsible for rating each candidate.

### Instrument

This study made use of the Bahrain Defence Force Instrument for professionalism, communication, and collaboration (BDF/PCC). It was established using several factors: the physician achievement review instrument (PAR) [12, 13]; the Maastricht list for history-taking and advice scoring instrument (MAAS-Global) [14], the Calgary-Cambridge tool, which measures communication abilities [15], the Sheffield peer review assessment tool (SPRAT) [1], the assessment of interprofessional team collaboration scale (AITCS) [16], and the opinions of specialists. The instrument focusses on the evaluation of professionalism, collaboration, and communication skills.

Previous studies were used to establish validity (face and content) for the BDF/PCC instrument [11]. It included 39 items, 15 of which measured professionalism, 13 of which to measured communication skills, and 11 of which measured collaboration. It was designed such that various groups of people, such as interns, consultants, senior medical colleagues, and coworkers, could all use it. It used a 5-point response scale, such that (1) meant “among the worst”; (2) meant “bottom half”; (3) meant “average”; (4) meant “top half”; and (5) meant “among the best”. There was also an option to provide “unable to assess” (UA) as a response.

### Statistical analysis

This study used several statistical analyses to answer the research questions. Mean and standard deviation were calculated for the total responses for each participant to determine who scored the highest. To ascertain the level of feasibility of the BDF/PCC instrument, we used both the rate of response and the number of responders necessary to obtain reliable results [1, 13].

To find the appropriate groupings of items on the survey, explanatory factor analysis was used. For each survey item, a factor was assigned, and it was given a loading factor equal or greater than 0.40. Whenever an item was cross-loaded (that is, loaded on 2 or more factors), it was given to the highest among the factors it was loaded on. To determine how many factors to extract, the Kaiser rule was used (that is, eigenvalues > 1.0). If an item was loaded on more than one factor (cross-loading), the item was assigned to the highest factor where it was loaded. The number of factors to be extracted was based on the Kaiser rule that eigenvalues are greater than 1.0 [17].

It was also necessary to determine how homogeneous each composite scale was. To do so, we calculated item-total correlations, with corrections for overlap [18]. An item was considered to measure the same construct as other composite scale items if and only if its total correlation coefficient was 0.3 or higher. We also used Pearson’s correlation coefficient for estimating inter-scale correlations, to find how much the scales overlapped [19].

To determine internal consistency and reliability, Cronbach’s coefficient—which is a common way of evaluating internal consistency—was used for each factor and each scale individually [18]. Next, a generalizability analysis was used to find the Ep^2^ and to make sure that enough questions were given and enough evaluators were used for there to be stable and accurate data for every intern. Previous studies showed that if Ep^2^ is 0.70 or higher, the data are stable; otherwise, there must be more items on the list or more responders in order to obtain adequate stability [11, 20].

### Responders

The responders for this study were organized into three groups: nurses, physicians, and fellow students. In order to be eligible to be a responder, they needed to have spent at least 1 or 2 months working alongside the graduate. Participants were asked to select eight individuals from each category, and the investigators randomly chose five out of these eight individuals, so that five individuals from each of the three above-mentioned groups rated each respondent. Different interns had different numbers of observers, and this difference was determined how many raters’ responses there were.

## Results

Our multisource feedback process achieved a response rate of only 16.5%, including 10 female and 6 male graduates. This low response rate may indicate that such a process is not ideal for use in selecting graduates for the interns’ award. The participants responded to most of the questionnaire’s questions.

Out of the 16 participants, the highest score was for a male graduate, who achieved 4.77 out of 5 as a mean rating. The second highest was a female whose total mean rating was 4.74. The lowest participant scored low on all 3 domains, giving a total mean rating of 3.54 out of 5 (Table 1).

**Table 1:** Number of observers and the mean score for knowledge, professionalism, communication skills and collaboration for the interns

Doctor ID#	Proposed total number of observer	Actual number of observers	Total mean score	Mean score in professionalism	Mean score in communication	Mean score in collaboration
1.	15	9	4.28	4.53	3.82	4.49
2.	15	14	4.04	4.02	4.11	4.00
3.	15	12	3.95	4.61	3.72	3.32
4.	15	13	4.74	4.95	4.67	4.55
5.	15	8	4.77	4.76	4.75	4.80
6.	15	9	4.48	4.71	4.40	4.27
7.	15	9	4.42	4.51	4.19	4.57
8.	15	8	3.78	4.32	3.51	3.36
9.	15	13	4.57	4.71	4.35	4.65
10.	15	12	4.56	4.70	4.37	4.61
11.	15	5	4.05	4.40	4.03	3.59
12.	15	15	3.54	3.99	2.98	3.59
13.	15	11	4.63	4.76	4.40	4.71
14.	15	15	4.53	4.47	4.54	4.61
15.	15	13	4.71	4.46	4.44	4.70
16.	15	9	4.24	4.28	4.20	4.23

First quartile (25th percentile) = 4.07, second quartile (50th percentile) = 4.19, third quartile (75th percentile) = 4.31

We found that the BDF/PCC instrument was suitable for factor analysis (KMO = 0.895; Bartlett test significant, p < 0.00). The response data from the questionnaire could therefore be decomposed into three factors—professionalism, communication, and collaboration—which accounted for 79.3% of the total variance.

Cronbach’s α coefficient for reliability and internal consistency was used to determine that BDF/PCC exhibits high levels of internal consistency, with Cronbach’s coefficient α equal to 0.98. For the factors, or subscales, within the questionnaire, there were also high levels of internal consistency and reliability, with Cronbach’s α coefficient greater or equal to 0.93. We replicated a previous D study to estimate the EP^2^ for up to ten raters, and determined that 1 assessor resulted in an EP^2^ value of 0.30; 8 assessors resulted in an EP^2^ value of 0.78; and 10 assessors resulted in an EP^2^ value of 0.81 [20] (Table 2).

**Table 2:** Descriptive statistics, item analysis, correlated item-total correlation and exploratory factor analysis

Q		N	M	SD	%UA	Correlated item-total correlation	Factors identified by factor analysis		
							Colla	Comm	Profe
Q1	Maintains confidentiality of patients	173	4.73	0.63	2.80	0.70		0.74	
Q2	Recognizes boundaries when dealing with other physicians	174	4.71	0.68	2.20	0.71	0.72		
Q3	Recognizes boundaries when dealing with other health care professionals	174	4.74	0.63	2.20	0.70	0.72		
Q4	Shows professional and ethical behavior	173	4.80	0.59	2.80	0.57	0.71		
Q5	Is punctual, and performs tasks in a time-appropriate manner	174	4.78	0.60	2.20	0.57	0.82		
Q6	Is able to handle situations in a professional manner and exhibits self-control, avoiding emotional outbursts in stressful situations	170	4.54	0.73	4.50	0.76			0.71
Q7	Respects patient’s autonomy and right to be involved in his/her own management	165	4.73	0.66	7.30	0.70		0.78	
Q8	Is reliable and responsible when preforming his duties	173	4.79	0.57	2.80	0.71	0.70		
Q9	Is honest, and handles his/her duties in a dignified manner	173	4.83	0.55	2.80	0.77	0.62		
Q10	Accepts constructive criticism and develops goals for improvement	164	4.59	0.80	7.90	0.71		0.67	
Q11	Respects cultural, individual and role differences including age, gender, race, religion, disability, language, sexual orientation, and socioeconomic status	173	4.75	0.63	2.80	0.70		0.76	
Q12	Follows institutional policies and procedures	176	4.70	0.66	1.10	0.51		0.70	
Q13	Arrives on time to scheduled appointments and hospital activities	168	4.72	0.68	5.60	0.71	0.81		
Q14	Manages healthcare resources efficiently	160	4.58	0.73	10.10	0.66		0.58	
Q15	Leads with respect and fair treatment of colleagues	169	4.69	0.66	5.10	0.68	0.56		
Q16	Communicates efficiently and in a clear, understandable fashion with colleagues within his/her team	173	4.79	0.57	2.80	0.73		0.57	
Q17	Communicates efficiently and in a clear, understandable, and compassionate way with patients	170	4.76	0.60	3.90	0.73		0.62	
Q18	Allows the patient to elaborate about his condition	162	4.67	0.67	9.00	0.74		0.70	
Q19	Communicates efficiently and in a clear, understandable, and compassionate way with patient’s families	160	4.74	0.56	10.1	0.62		0.56	
Q20	Communicates clearly and effectively with other healthcare workers, e.g. nurses	170	4.69	0.62	4.50	0.74	0.61		
Q21	Explains what is being done for the patient during examination or procedures	157	4.67	0.70	11.8	0.66		0.61	
Q22	Communicates purpose and results of investigations to patients well	150	4.65	0.67	15.7	0.64		0.57	
Q23	Follows up appropriately and in a timely manner on patients’ hospital course	160	4.66	0.69	10.1	0.66			0.69
Q24	Communicates management options to patients in a clear, understandable way, taking into account the patients’ opinion	150	4.69	0.66	15.7	0.67			0.69
Q25	Displays empathy in dealing with patients by eye contact and verbal responses	156	4.72	0.65	12.4	0.70		0.68	
Q26	Summarizes the information given for the patient in small quantities, with concrete explanations, and understandable language	155	4.67	0.65	12.9	0.66			0.74
Q27	Maintains calm in emergency situations, in order to communicate information clearly to his/her seniors	152	4.57	0.72	14.6	0.71			0.76
Q28	Communicates accurate patient information to physicians from other departments when required to do so	161	4.70	0.61	9.60	0.58			0.69
Q29	Manages to work well as part of a healthcare team	165	4.70	0.67	7.30	0.61	0.76		
Q30	Facilitates the learning of medical colleagues and co-workers	159	4.70	0.68	10.7	0.59		0.54	
Q31	Collaborates well with nurses and other healthcare workers	162	4.73	0.63	9.00	0.69	0.68		
Q32	Concerned about the safety of patients and co-workers	166	4.63	0.69	6.70	0.70			0.55
Q33	Coordinates patient care efficiently	164	4.71	0.63	7.90	0.67			0.62
Q34	Collaborates with other healthcare workers in order to achieve optimal patient care	168	4.70	0.66	5.60	0.58	0.65		
Q35	Participates in a system of call in order to provide care for patients	164	4.67	0.71	7.90	0.57	0.68		
Q36	Provides appropriate guidance and help to team members on regular bases	163	4.63	0.71	8.40	0.56			0.72
Q37	Takes an extra work, when appropriate, to help the team	165	4.67	0.74	7.30	0.61	0.70		
Q38	Enables the team to achieve agreements for team process and collaborative completion of assignment	162	4.63	0.72	9.00	0.62			0.67
Q39	Participates fully in collaborative process and fulfilled team agreements	167	4.68	0.68	6.20	0.68	0.62		

*N* number, *M* mean, *SD* standard deviation, *UA* unable to assess, *Comm* communication skills, *Colla* collaboration, *Profe* professionalism

### Discussion

In this study, we introduced a new method to select graduates for Medical School awards. As far as we know, no past studies have used the MSF process to choose graduates for college awards. Although this study found that MSF is a way to valid and reliable process for such a task, our low response rates mean that we cannot claim it as a feasible method.

Multisource feedback, which is also called 360° evaluation, has become a widely used way to evaluate trainees across diverse fields and for various reasons [21]. Furthermore, this study found that the MSF process was a valid and reliable way to assess university students’ professionalism, collaboration, and communication skills. Since few raters are required to obtain reliable evaluation, the MSF process is shown to have high feasibility; however, this feasibility is undermined by our low response rates.

Three composite scales were obtained through this study’s exploratory factor analysis: professionalism, collaboration, and communication skills. Using factor analysis, it was shown that the questionnaire could be divided into three factor solutions, which accounted for a measure of total variance amounting to 79.3%, which shows that the instrument has high construct validity.

The validity of the MSF process is supported by the fact that it has high levels of reliability, as well as item-total and inter-scale correlation, all within predefined limits. With such strong evidence, medical institutions may feel confident in selecting the best graduates for the award because the results obtained using the MSF process were both reliable and valid.

Our findings may be preferable to other previously used methods to select the best graduates for the university award such as letters of recommendation, honor grades, and other factors. This is because the majority of the other methods do not include direct observation of the applicants and, therefore, may be less useful indicators for particular success-predicting behaviors [22].

Our study examined a wide range of applicants’ variables, such as professionalism, communication skills, and collaboration. Additionally, the use of the MSF process on its own strengthens and supports the results of the study. Other methods may be easy to use and may have encouraged many candidates to participate, but they would have uncertain reliability. The number of raters and the psychometric properties of the instruments used in the selection of graduates provide strong evidence about the quality of the selection process [23–25].

Another advantage of MSF is that if the individual being assessed believes that the process is a trustworthy and correct method of self-improvement, they will likely use it to make changes and improve in the future. This will also help graduates pay more attention to their future performance in areas requiring improvement [3, 26].

In a study of family physicians, it was found that 61% of the 113 participating physicians changed or planned to change their practice based on the feedback that the program director gave after the MSF process [27]. As a general rule, the results of this study show that the only ones who used the results of the feedback to work towards self-improvement were those who believed the process was correct and trustworthy [27].

One of the important limitations of this study was the low response rates, which indicate that the MSF process used to select the best graduates is not feasible. Future studies may be useful for further examining the MSF process’s feasibility in selecting of the best interns for the university awards.

## Conclusion

“This study demonstrated that the MSF tool can be used as a valid and reliable method to select candidates for students’ award in higher education. The results of this study can be used by many institutions to enhance their selection methods for graduate awards. However, the low response rate seems to suggest that although the use of the MSF is promising, it may not be feasible. Therefore, to demonstrate the feasibility of this tool future studies are recommended to further examine the use of the MSF in selecting candidates for awards”.

## Abbreviations

MSF: multisource feedback
RCSI: Royal College of Surgeons in Ireland
BDF/PCC: Bahrain Defence Force/Professionalism Communication Collaboration
